# Determination and mitigation of the uncertainty of neutron diffraction measurements of residual strain in large-grained polycrystalline material

**DOI:** 10.1107/S1600576715002757

**Published:** 2015-03-12

**Authors:** Tom M. Holden, Yeli Traore, Jon James, Joe Kelleher, P. John Bouchard

**Affiliations:** a Engineering and Innovation, The Open University, Walton Hall, Milton Keynes MK7 6AA, UK

**Keywords:** measurement uncertainty, neutron diffraction, residual strain, polycrystalline materials

## Abstract

For large-grained samples it is advantageous to perform pairs of neutron diffraction measurements at the same spatial location but rotated 180° around the geometric centre of the gauge volume as a means of minimizing the scatter coming from the random positioning of grains within the gauge volume.

## 1. Introduction   

The measurement of residual strains by neutron diffraction in large-grained polycrystalline material is inherently inaccurate. The reason for this, surmised for many years (Hutchings *et al.*, 2005 ▶; Ohms, 2013 ▶), is the random positioning of the few diffracting grains in the gauge volume, the region of overlap between the incident and diffracted beams. Calibration of the neutron diffraction instrument makes use of a fine-grained standard powder whose centre of diffraction coincides with the geometric centre of the gauge volume in a perfect setup. In large-grained polycrystalline materials used for engineering applications there is every reason to expect that large grains will be spatially offset from the geometric centre, but in a random way. A systematic error is therefore introduced, analogous to a partly filled gauge volume, that shifts the diffraction pattern sometimes above, sometimes below and sometimes close to the correct result. The consequence is that the scatter in measured lattice parameter from point to point in the material can exceed the expected fitting precision of the diffraction peaks, by a margin as much as an order of magnitude. In analysing neutron diffraction data from an international round robin for a weld bead-on-plate test specimen, Wimpory *et al.* (2009 ▶) suspected that underestimation of the uncertainty in stress determination originated from grain size issues. Subsequently, Wimpory *et al.* (2010 ▶) developed a simple model quantifying the extra uncertainty that has to be added because of grain-size effects when there are an insufficient number of diffracting grains within the gauge volume. Errors arising from large grains can be mitigated by selecting a relatively large gauge volume or by ‘rocking’ the specimen to increase the number of diffracting grains sampled, but these approaches become less effective as the grain size increases (Ohms, 2013 ▶).

Recently, in the course of a neutron diffraction residual stress measurement on a thick-walled pipe, of outer diameter 352 mm and thickness 40 mm, containing a dissimilar metal girth weld, the opportunity arose to make multiple stress-free reference lattice parameter measurements in three materials of different grain sizes: a fine-grained ferritic steel (16MND5), a large-grained austenitic stainless steel (Type 316L) with a grain size range up to 1000 µm and an average grain size of 313 µm, and nickel-based Alloy-52 weld metal with a similar grain size and complex columnar structure. These lattice parameter measurements allowed the expected standard deviation to be determined for the large-grained samples as well as for a control sample of ferritic steel with small grain size.

## 2. Method   

The experiments were carried out on the ENGIN-X neutron time-of-flight diffractometer at the ISIS spallation neutron source at the Rutherford Appleton Laboratory. The incident beam was defined by a cadmium mask 4 mm high and 4 mm wide. The diffracted beam passed through 4 mm radial collimators to two counter banks at ±90°, which span ±10° in the horizontal plane and ±15° in the vertical plane. The time-of-flight spectra were analysed using the Rietveld refinement method of Von Dreele *et al.* (1982 ▶), giving a single lattice parameter result for each measurement.

Reference lattice parameters were measured in small cylinders of diameter 6.2 mm and length 40 mm that had been extracted from the pipe weldment by wire electro-discharge machining (EDM). The axes of the reference cylinders sampling the stainless steel, ferritic steel and Alloy-52 materials were aligned with the pipe radial direction. Narrow circumferential grooves were cut by wire EDM at 6 mm intervals along each reference cylinder. The depth of the grooves was controlled to leave a 2 mm square cross section connecting ligament. This approach reduced the residual stress acting along the axis of the reference cylinder to an insignificant level. The magnitude of residual stresses remaining in the 6 mm reference cylinders was assessed to be low on the basis of numerical studies of Repper *et al.* (2012 ▶) and application of the closed form analysis described by Traoré *et al.* (2013 ▶), assuming an initial residual stress field with a wavelength of the order of the pipe thickness.

**Figure 1:**
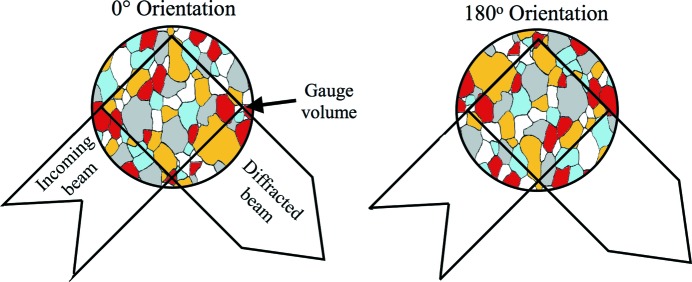
Schematic representation showing how the positions of grains change with respect to the centre of the gauge volume upon rotation from 0° orientation through 180° about the geometric centre. The different colours represent families of grains where the normals to common crystallographic planes are aligned in the same direction (*i.e.* families of diffracting grains).

The reference cylinders were mounted vertically in a three-jaw chuck on the sample table, allowing measurements in the pipe axial-hoop plane at six positions midway between the cuts. In the case of the 16MND5 ferritic steel reference cylinder, five measurements were made along the length and a single measurement in the centre of a stainless steel cladding (∼7.5mm thick) adjacent to the inside of the pipe. Measurements were made at 0 and 180°, corresponding to the pipe hoop direction, and at 90 and 270° for the pipe axial direction. In two measurements 180° apart, grains offset to the right of the centre of the gauge volume will move to the left of centre, as the cartoon in Fig. 1 ▶ shows. For this reason, the systematic error in the diffraction incurred at 0° tends to reverse sign at 180°. Averaging these pairs of measurements will tend to cancel out the systematic errors, though it is unlikely to reduce them to zero owing, for example, to variations in intensity across the neutron beam. It is reasonable to expect that there will be no strong radial alloy concentration gradient in any of the materials and also that the two diffracted beam counter banks of the ENGIN-X instrument will give equally acceptable values of lattice parameter. This latter assumption can be tested for the fine-grained ferrite case. Thus, 48 equivalent measurements of lattice parameter were made at six radial locations, four angles and two counters. These permitted estimates to be made of the standard deviation resulting from large grains.

## 3. Results   

**Figure 2:**
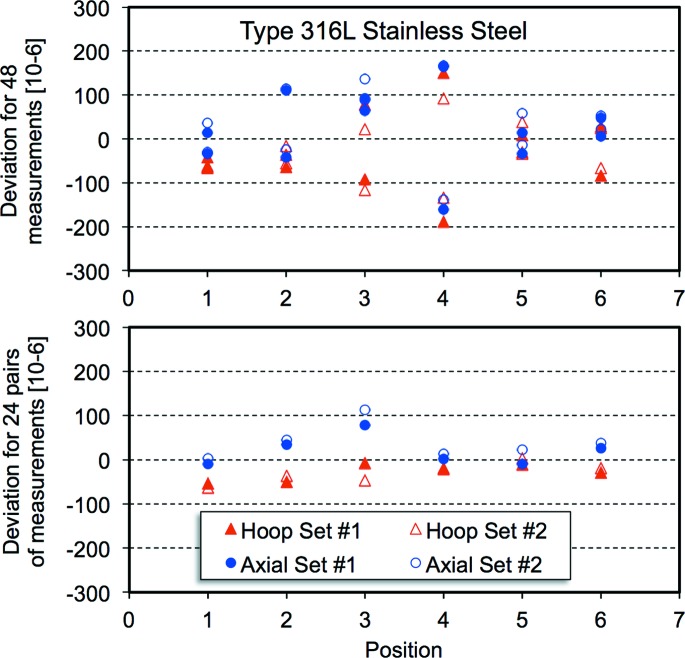
Deviations from the average lattice parameter, expressed in the form Δ*a*/*a*, for 48 measurements (top) and 24 pairs (bottom) for the Type 316L austenitic stainless steel. The average lattice parameter was 3.59500 Å and the standard deviations, expressed as a strain, were 84 and 42 × 10^−6^, respectively.

**Figure 3:**
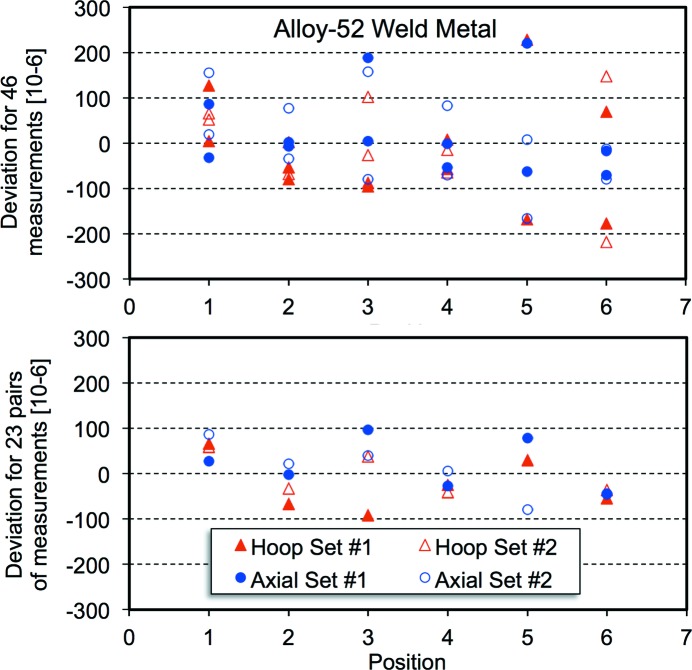
Deviations from the average lattice parameter, expressed in the form Δ*a*/*a*, for 46 measurements (top) and 23 pairs (bottom) for the Alloy-52 weld metal. The average lattice parameter was 3.57947 Å and the standard deviations, expressed as a strain, were 103 and 55 × 10^−6^, respectively.

**Figure 4:**
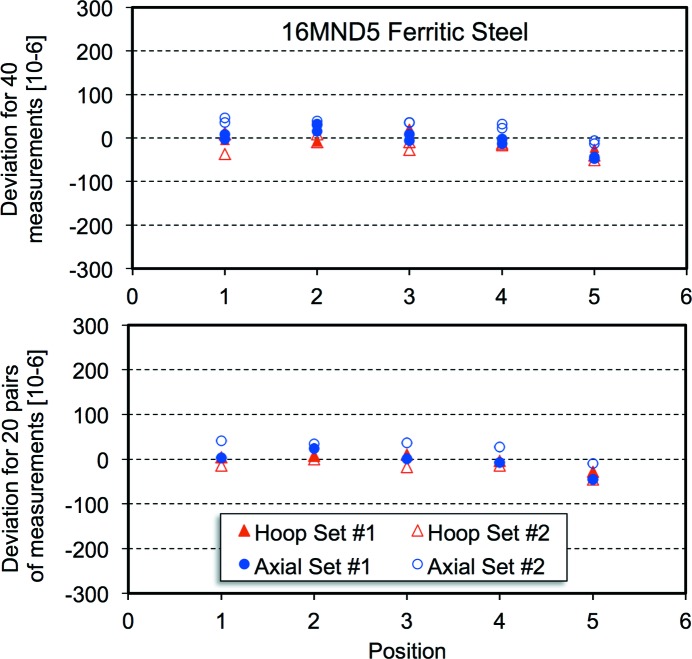
Deviations from the average lattice parameter, expressed in the form Δ*a*/*a*, for 40 measurements (top) and 20 pairs (bottom) for the 16MND5 ferritic steel. The average lattice parameter was 2.86772 Å and the standard deviations, expressed as a strain, were 26 and 25 × 10^−6^, but included a slight difference between collector bank 1 and collector bank 2 as well as a variation through wall of about −40 × 10^−6^.

Fig. 2 ▶ shows the distribution of offsets about the average value of the lattice parameter, expressed in the form Δ*a*/*a*, as a function of position along the reference cylinder for all 48 measurements for the large-grained 316L austenitic stainless steel and the 24 averages of pairs of values. The average lattice parameter and standard deviation are noted. The standard deviation is reduced by 50% for the pairs. Fig. 3 ▶ presents similar results for the Alloy-52 weld metal, which had a columnar grain structure. Fig. 4 ▶ illustrates the distribution of offsets about the average lattice parameter for the 40 measurements of strain for the ferritic steel. In this case, the standard deviation only decreases by about 5%. A minor difference between the two counter banks [24 (12) × 10^−6^] can be discerned, but this is far less than the offsets for the large-grained material. For the ferritic material there is a maximum variation in lattice parameter, expressed as a strain between the outside and inside surfaces of the pipe of 40 × 10^−6^.

## 4. Discussion   

In the calculation of residual strain for the intact sample weldment (*i.e.* the pipe/dissimilar metal girth weld) there will be four contributions to the standard error: two from the fitting errors to the spectrum, δ_fit,sample_ and δ_fit,ref_, one from the large grains (lg) for the reference lattice parameter, δ_ref,lg_, and one from the large grains in the intact sample, δ_sample,lg_, which can be added in quadrature. δ_sample,lg_ in this case would be the standard deviation determined from the 48 measures of random grain offsets, since measurements cannot be made at 0 and 180° because of the geometry of the bulky sample and experimental time constraints. On the other hand δ_ref,lg_ can be taken as the standard deviation of the 24 pairs, since measurements can be readily made at 0 and 180° on the relatively small coupons. In general, δ_fit,sample_ only makes a significant contribution to the error in the one or two cases where the path length through the material is long and the spectrum weak.

For the large-grained samples, the 48 measurements gave a standard deviation that was about eight times the fitting error. For the small-grained ferritic sample, the standard deviation of pairs of measurements was on average only 10 (8) ×10^−6^, that is, about 50% greater than the fitting error to the spectrum. Thus, the latter is seen to be a reasonable estimate of the error in the absence of other information, although it has always been recognized as being a lower limit on the standard deviation of a diffraction measurement.

The same considerations apply to the errors in angular dispersive neutron diffraction. Time-of-flight diffraction, however, does have the advantage of a far larger counter area, thus collecting data from more crystallites as well as averaging over the several peaks in the diffraction spectrum, in this case {111}, {002}, {220} and {113} for austenitic material and {110}, {002}, {112} and {222} for the ferritic material. Continual rotation of the reference sample in the axial hoop plane would have given a satisfactory reference lattice parameter, but the information on the standard deviation coming from large grains is hidden in the line width. In the case of high-energy X-ray synchrotron measurements, it could be advantageous to defocus the beam to carry out the same kind of analysis.

## 5. Conclusions   

In the case of a large-grained sample it is advantageous to perform pairs of measurements at the same spatial location but rotated 180° around the geometric centre of the gauge volume as a means of minimizing the standard deviation and scatter coming from the random positioning of grains within the gauge volume. If a sufficient number of equivalent measures of lattice parameter can be made in the reference samples, the standard deviation associated with large grains can be assessed for the sample. For small-grained samples, such as the ferritic material in this case, the fitting error to the spectrum may be a reasonable estimate of the standard deviation in the absence of other information.
